# Association of Early Nephrology Consultation with Mortality in Patients with Severe Hyponatremia

**DOI:** 10.34067/KID.0000001197

**Published:** 2026-04-14

**Authors:** Akira Nakamura, Takashin Nakayama, Tatsuhiko Azegami, Kyosei Nakamura, Hiroaki Miyauchi, Shinnosuke Sugihara, Ryunosuke Mitsuno, Norifumi Yoshimoto, Akihito Hishikawa, Aika Hagiwara, Motoaki Komatsu, Kaori Hayashi

**Affiliations:** 1 Division of Nephrology, Endocrinology, and Metabolism, Department of Internal Medicine, Keio University School of Medicine, Tokyo, Japan; 2 Department of Nephrology, Tokyo Saiseikai Central Hospital, Tokyo, Japan

**Keywords:** hyponatremia, mortality

## Abstract

**Key Points:**

- Early nephrology consultation was associated with lower in-hospital and 30-day mortality in patients with severe hyponatremia.
- Collaboration between nephrologists and other specialties is essential for optimal hyponatremia management.

**Background:**

Hyponatremia is the most common electrolyte disorder and is encountered by physicians across multiple specialties. Its management requires precise correction of the serum sodium level, which may benefit from the involvement of specialists, particularly nephrologists. This study aimed to evaluate the impact of nephrology consultation on mortality.

**Methods:**

This retrospective observational study included patients with severe hyponatremia (serum sodium level ≤120 mEq/L) at admission or during hospitalization between January 2014 and June 2024 in two tertiary care centers. Nephrology consultation was defined as any therapeutic involvement by nephrologists within 24 hours of the onset of severe hyponatremia. The association of nephrology consultation with in-hospital and 30-day mortality was assessed using inverse probability of treatment weighting based on propensity scores.

**Results:**

A total of 942 patients were included in the analysis, of whom 342 (36%) received nephrology consultation and 600 (64%) did not. The median (interquartile range) age was 73 (62–82) years, 430 (46%) patients were female, and serum sodium levels were 118 (116–119) mEq/L. In weighted logistic regression analyses, nephrology consultation was associated with lower in-hospital and 30-day mortality (odds ratio, 0.55; 95% confidence interval, 0.37 to 0.83 and odds ratio, 0.62; 95% confidence interval, 0.40 to 0.96, respectively). Osmotic demyelination syndrome occurred in three patients, with no significant difference in incidence between the two groups (*P* = 0.27).

**Conclusions:**

Early nephrology consultation was associated with lower in-hospital and 30-day mortality in patients with severe hyponatremia, underscoring the importance of collaboration between nephrologists and other specialties in its management.

## Visual Abstract

**Figure:**
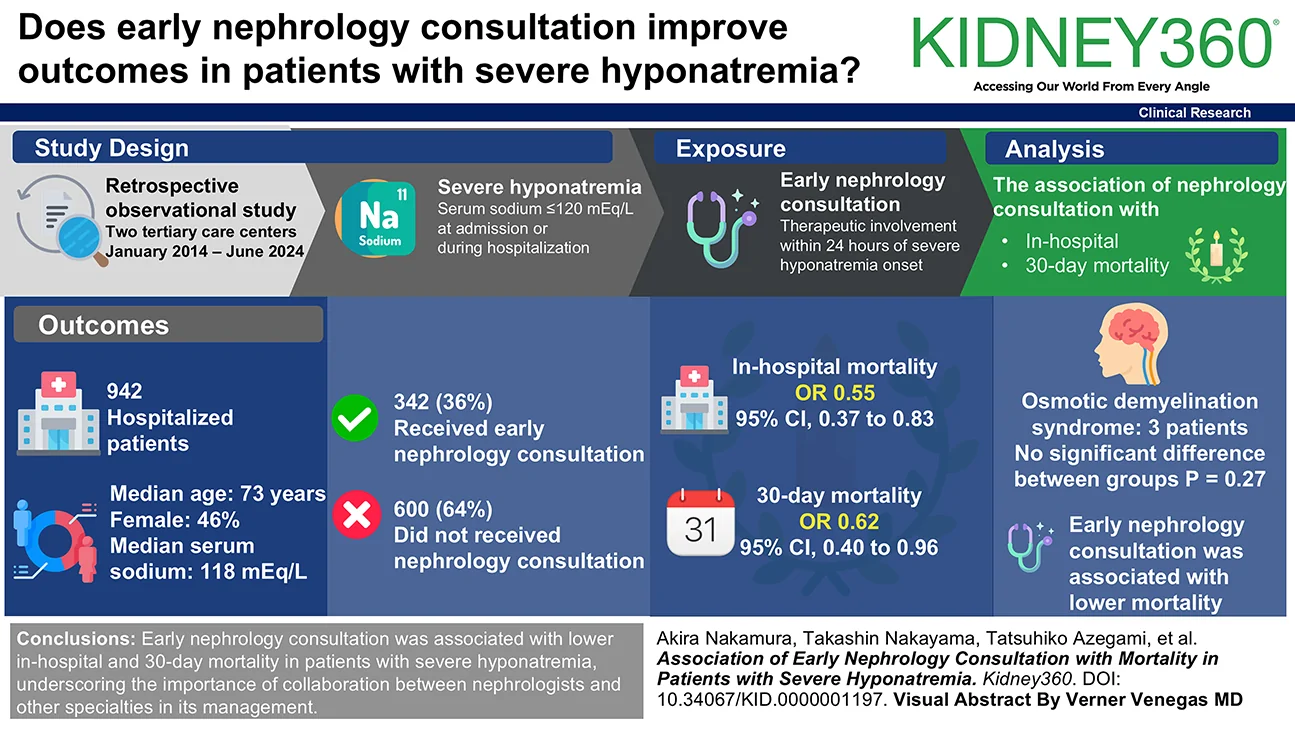

## Introduction

Hyponatremia is the most common electrolyte disorder with a prevalence of 30% among hospitalized patients.^1,2^ Advanced age, CKD, and heart failure are major risk factors for hyponatremia, and because the prevalence of these underlying diseases continues to rise, the number of patients developing hyponatremia is also expected to increase.^3–5^ In severe cases, hyponatremia presents with a broad spectrum of neurological and systemic symptoms and is associated with increased mortality.^6,7^ Given these considerations, hyponatremia remains a critical clinical concern.^2,6^

The management of hyponatremia presents significant clinical challenges. Excessively rapid correction of serum sodium can lead to osmotic demyelination syndrome (ODS), a serious and potentially irreversible neurological complication.^8,9^ To prevent this, current guidelines recommend limiting the correction rate to no more than 10–12 mEq/L over 24 hours.^3,4^ On the other hand, in patients with neurological manifestations, more rapid correction is often necessary to relieve acute symptoms.^3–5,10,11^ Indeed, concerns have been raised about the potential adverse effects of overly slow correction on clinical outcomes.^12–15^ These conflicting imperatives highlight the difficulty in determining an appropriate correction rate. Achieving safe and effective correction requires individualized assessment and therapeutic strategies tailored to each patient's clinical context.^3–5,10^

Given its frequent incidence and diverse clinical presentations, hyponatremia is often managed by non-nephrology specialties at the initial point of care. However, considering the complexity of sodium correction and its potential risks, timely involvement of nephrologists may play a critical role in optimizing patient outcomes. Although timely nephrology consultation has been reported to improve outcomes in CKD and AKI, its role in the management and prognosis of patients with hyponatremia remains insufficiently explored.^12,16–18^ Therefore, we aimed to evaluate whether early nephrology consultation contributes to better clinical outcomes in patients with severe hyponatremia.

## Methods

### Study Population

We conducted a retrospective cohort study of patients with severe hyponatremia (serum sodium ≤120 mEq/L), identified either at admission or during hospitalization, between January 2014 and June 2024 at Keio University Hospital and Tokyo Saiseikai Central Hospital.^18,19^ The exclusion criteria were as follows: (*1*) obvious measurement errors; (*2*) younger than 18 years; (*3*) blood glucose >500 mg/dl; (*4*) management without hospitalization; (*5*) absence of follow-up sodium measurements. For patients with recurrent severe hyponatremia, only the first episode was considered. This study and all associated protocols were reviewed and approved by the Ethics Committee of Keio University School of Medicine (approval number: 20241180) and conducted in accordance with the principles of the Declaration of Helsinki. Informed consent was obtained through an opt-out method through institutional websites.

### Data Collection

Clinical information at hyponatremia onset—including age, sex, height, weight, alcohol abuse, do-not-resuscitate (DNR) order as a potential indicator of treatment limitation, clinical setting (inpatient or outpatient), presence of sepsis, comorbidities, and concurrent medications—was collected from electronic medical records. DNR order was collected as a surrogate marker of treatment limitation and overall illness severity, which could influence both the likelihood of nephrology consultation and mortality risk. The comorbidities assessed included diabetes mellitus, heart failure, stroke, cirrhosis, chronic obstructive pulmonary disease, dementia, schizophrenia, malignancy, and metastatic tumor. The Charlson Comorbidity Index (CCI) was also calculated to quantify overall comorbidity burden. Care unit during the initial 24 hours (critical care units or general wards) was also recorded. Laboratory data at the onset of hyponatremia were obtained, including serum and urinary sodium and potassium levels, serum albumin and creatinine levels, and hemoglobin. Follow-up serum sodium levels were also recorded. In addition, information on the management of hyponatremia was obtained, including the use of hypertonic saline and V2 receptor antagonists and the frequency of serum and urinary sodium measurements. In clinical practice in Japan, commercially available 3% sodium chloride solution is not routinely used. Instead, concentrated sodium chloride (*e.g*., 10%) is often added to 0.9% saline to achieve the sodium concentration, typically resulting in hypertonic saline solutions with concentrations up to 3%. The eGFR was calculated using the following equation: 194×Serum creatinine^−1.094^×Age^−0.287^ (×0.739 if female).^20^

### Serum Sodium Levels

For each study participant, the 24-hour serum sodium correction was calculated. The baseline time point was defined as the moment when the serum sodium level was first recorded ≤120 mEq/L (Na [0]). Because serum sodium levels were not routinely measured exactly 24 hours after this baseline time point, the serum sodium level at 24 hours (Na [24]) was estimated by linear interpolation using the closest measured values before and after 24 hours from the onset of severe hyponatremia. The calculation was performed using the following formula: Na (24)=Na^a^+([Na^b^−Na^a^]×[24−T^a^]/[T^b^−T^a^]), where Na^a^ and T^a^ represent the closest serum sodium levels and time points before 24 hours, respectively, and Na^b^ and T^b^ are those after 24 hours, respectively.^13,18,19^ The change in serum sodium over 24 hours was then calculated as Na (24)—Na (0). To evaluate the effect of sodium correction rate, patients were stratified into three categories on the basis of the increase in serum sodium concentration within 24 hours: slow correction (<6 mEq/L), optimal correction (6–10 mEq/L), and fast correction (>10 mEq/L), as previously described.^13,18^

### Exposure and Outcomes

Nephrology consultation was defined as the presence of a nephrologist note or clinical order within 24 hours of hyponatremia onset in the electronic medical record, irrespective of primary or consultative role. All other cases were classified as non-nephrology consultation. The 24-hour threshold was based on prior studies in severe hyponatremia, demonstrating that the rate of serum sodium correction within the first 24 hours is associated with mortality and neurological complications,^13^ as well as studies in AKI showing that early nephrology consultation is associated with improved outcomes compared with delayed consultation.^17,21^ The primary outcomes were in-hospital and 30-day mortality. In addition, we compared changes in serum sodium levels within 24 hours, length of hospital stay, and the incidence of ODS.

### Statistical Analyses

Clinical characteristics, management strategies, and outcomes of the participants were stratified according to the presence or absence of nephrology consultation (nephrology consultation versus non-nephrology consultation). Continuous variables are presented as medians with interquartile ranges and categorical variables as numbers (percentages). Differences in continuous variables between the two groups were assessed using the Mann–Whitney *U* test. For categorical variables, differences were examined using the chi-squared test. To evaluate the effect of nephrology consultation on in-hospital and 30-day mortality, logistic regression analyses were performed to determine odds ratios (ORs) with 95% confidence intervals (CIs). From a clinical perspective and previous knowledge, the following variables were selected as covariates for the multivariable models: age, sex, body mass index, CCI, DNR order, clinical setting at onset (inpatient versus outpatient), eGFR, and serum sodium levels.^13,18^ To further address potential confounding, propensity scores representing the probability of receiving nephrology consultation were estimated using a logistic regression model that included all the above covariates. Stabilized inverse probability of treatment weighting (IPTW) on the basis of the propensity scores was then applied to create a weighted pseudo-population in which baseline characteristics were balanced between the nephrology consultation and non-nephrology consultation groups. Weighted logistic regression analyses were subsequently performed to estimate the association between nephrology consultation and mortality outcomes. Subsequently, we conducted subgroup analyses on the basis of age (75 or younger versus older than 75 years), sex (male versus female), CCI (≤3 versus >3), eGFR (≥60 versus <60 ml/min per 1.73 m^2^), and clinical setting at the onset of hyponatremia (outpatient versus inpatient). Propensity scores were calculated separately for each subgroup, and adjustments using IPTW were used accordingly.

To ensure the robustness of our findings, we performed six sensitivity analyses. First, to reflect more granular, disease-specific clinical conditions, we replaced the CCI with individual comorbid conditions (heart failure, cirrhosis, and metastatic tumor) and further adjusted for sepsis and additional laboratory parameters (albumin and hemoglobin). Second, an analysis excluding patients with DNR order was conducted. Third, we restricted the analyses to patients managed in general wards during the initial 24 hours. Fourth, we repeated the analysis after excluding patients with blood glucose >200 mg/dl. Fifth, missing data were handled using multiple imputation by chained equations with 20 iterations.^22^ Sixth, we conducted a sensitivity analysis in which the non-nephrology consultation group from the primary analysis was subdivided into delayed nephrology consultation (after 24 hours) and no nephrology consultation throughout hospitalization. This analysis was performed to evaluate whether the observed association with mortality was attributable to early nephrology consultation rather than to patient characteristics prompting consultation.

We performed all analyses using STATA version 18.0 (StataCorp LLC, College Station, TX). All reported *P* values were two-sided, and *P* values < 0.05 were considered statistically significant.

## Results

### Characteristics of the Study Population

**Figure 1:**
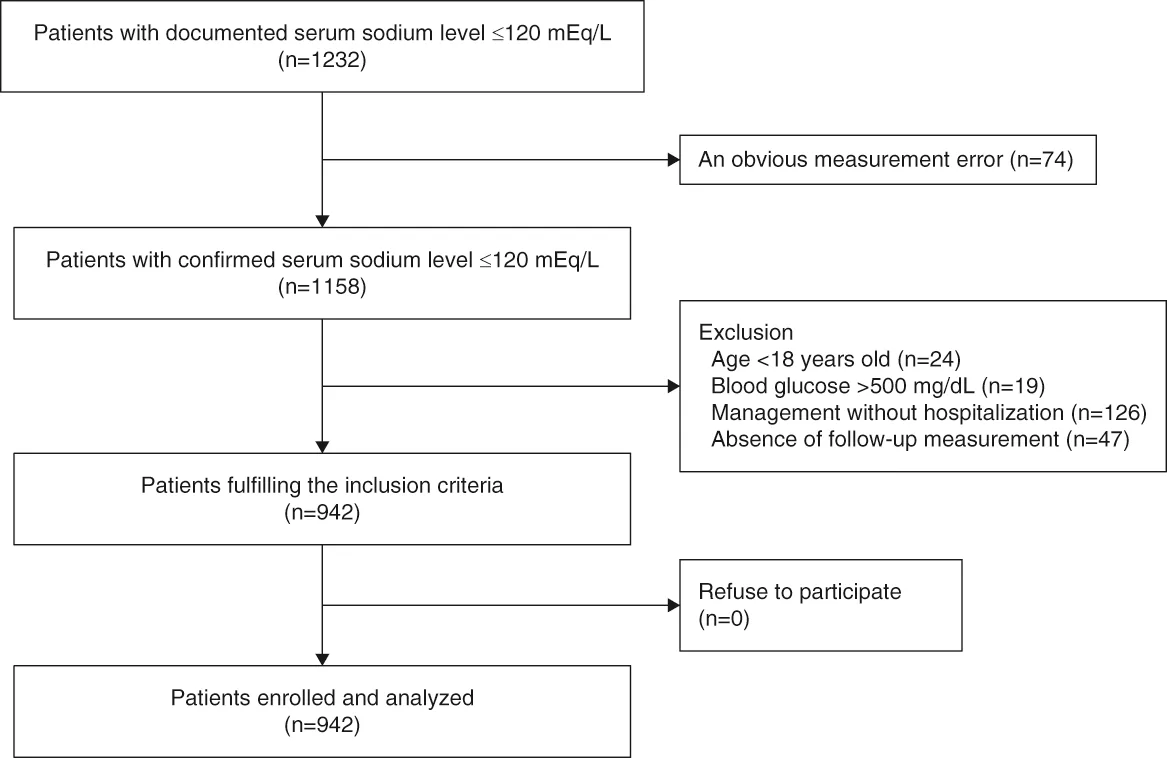
Flow chart of patient selection.

Among the 1232 patients with serum sodium levels ≤120 mEq/L, a total of 290 were excluded: 74 had obvious measurement errors, 24 were younger than 18 years, 19 had blood glucose levels >500 mg/dl, 126 were not admitted, and 47 had no follow-up measurement (Figure 1). Consequently, 942 patients were included in the final analysis.

Baseline characteristics are described in Table 1. The median age was 73 (62–82) years, 430 (46%) patients were female, and serum sodium levels were 118 (116–119) mEq/L. Of the entire cohort, 342 patients (36%) received nephrology consultation, while 600 (64%) did not. Compared with the non-nephrology consultation group, patients in the nephrology consultation group were less likely to have a DNR order (9% versus 15%), more likely to develop hyponatremia in the outpatient setting (59% versus 42%), and had a lower prevalence of cirrhosis (6% versus 10%). Regarding laboratory data, the nephrology consultation group exhibited lower serum sodium (117 versus 118 mEq/L) and potassium levels (4.3 versus 4.5 mEq/L), higher serum albumin levels (3.5 versus 3.1 g/dl), and reduced eGFR (73 versus 78 ml/min per 1.73 m^2^). Other baseline characteristics were comparable between the two groups. Table 2 summarizes the details of management of hyponatremia. Overall, 27% of patients received hypertonic saline, which was used more frequently in the nephrology consultation group than in the non-nephrology consultation group (40% versus 20%, *P* < 0.001). Intermittent bolus administration was more frequently employed in the nephrology consultation group (7% versus 1%, *P* < 0.001), which also underwent more frequent monitoring of serum sodium (three versus two measurements, *P* < 0.001) and urinary sodium levels (two versus one measurements, *P* < 0.001) within 24 hours.

**Table 1:** Characteristics of patients with severe hyponatremia stratified by nephrology consultation

Characteristics	Total (*N*=942)	Non-nephrology Consultation Group (*n*=600)	Nephrology Consultation Group (*n*=342)
Age, yr	73 (62–82)	73 (61–82)	74 (63–83)
Sex (female)	430 (46%)	265 (44%)	165 (48%)
BMI (*n*=857)	19.8 (17.5–22.6)	19.8 (17.4–22.6)	19.9 (17.5–22.2)
Alcohol abuse	102 (11%)	65 (11%)	37 (11%)
DNR order	120 (13%)	90 (15%)	30 (9%)
Setting at hyponatremia onset (outpatient)	454 (48%)	252 (42%)	202 (59%)
Care unit in the first 24 h (critical care unit)	120 (13%)	85 (14%)	35 (10%)
Sepsis	111 (12%)	78 (13%)	33 (10%)
**Comorbidities**			
Diabetes mellitus	232 (25%)	143 (24%)	89 (26%)
Heart failure	230 (24%)	140 (23%)	90 (26%)
Stroke	119 (13%)	78 (13%)	41 (12%)
Cirrhosis	84 (9%)	62 (10%)	22 (6%)
COPD	40 (4%)	28 (5%)	12 (4%)
Dementia	107 (11%)	70 (12%)	37 (11%)
Schizophrenia	14 (2%)	9 (2%)	5 (2%)
Malignancy	406 (43%)	261 (44%)	145 (42%)
Metastatic tumor	199 (21%)	131 (22%)	68 (20%)
CCI	3 (1–6)	3 (1–6)	3 (1–6)
**Medications**			
Loop diuretics	190 (20%)	124 (21%)	66 (19%)
Thiazide diuretics	66 (7%)	40 (7%)	26 (8%)
MRA	123 (13%)	84 (14%)	39 (11%)
NSAIDs	136 (14%)	84 (14%)	52 (15%)
Opioids	104 (11%)	68 (11%)	36 (11%)
Antiepileptic drugs	62 (7%)	46 (8%)	16 (5%)
Antipsychotic drugs	53 (6%)	32 (5%)	21 (6%)
Antidepressants	66 (7%)	38 (6%)	28 (8%)
**Admission laboratory data**			
Serum sodium, mEq/L	118 (116–119)	118 (117–120)	117 (115–119)
Serum albumin, g/dl	3.2 (2.6–3.8)	3.1 (2.5–3.6)	3.5 (2.8–4.0)
Serum potassium, mEq/L	4.5 (3.9–5.0)	4.5 (4.0–5.0)	4.3 (3.7–4.9)
Serum creatinine, mg/dl (*n*=941)	0.69 (0.51–1.11)	0.68 (0.51–1.01)	0.71 (0.50–1.66)
eGFR, ml/min per 1.73 m^2^ (*n*=941)	76 (46–104)	78 (50–106)	73 (28–103)
Hemoglobin, g/dl	11.1 (9.5–12.6)	11.0 (9.3–12.5)	11.1 (9.6–12.7)
Urinary sodium, mEq/L (*n*=598)	56 (27–100)	54 (25–96)	59 (30–106)
Urinary potassium, mEq/L (*n*=582)	30 (20–43)	31 (22–45)	29 (18–41)

Data are expressed as median (interquartile range) or number (percentage).

BMI, body mass index; CCI, Charlson Comorbidity Index; COPD, chronic obstructive pulmonary disease; DNR, do-not-resuscitate; MRA, mineralocorticoid receptor antagonist; NSAIDs, non-steroidal anti-inflammatory drugs.

**Table 2:** Management of patients with severe hyponatremia stratified by nephrology consultation

Management	Total (*N*=942)	Non-nephrology Consultation Group (*n*=600)	Nephrology Consultation Group (*n*=342)	*P* Value
**Hypertonic saline treatment**	258 (27%)	122 (20%)	136 (40%)	<0.001
Pattern of administration				
*Continuous infusion*	230 (24%)	117 (20%)	113 (33%)	<0.001
*Intermittent bolus*	28 (3%)	5 (1%)	23 (7%)	
V2 receptor antagonists	55 (6%)	31 (5%)	24 (7%)	0.24
**Number of measurements**				
Serum sodium	2 (2–3)	2 (2–3)	3 (2–5)	<0.001
Urinary sodium	1 (0–2)	1 (0–1)	2 (1–3)	<0.001

Data are expressed as median (interquartile range) or number (percentage). Hypertonic saline refers to saline solutions with a sodium concentration higher than 0.9%. The number of serum and urinary sodium measurements refers to measurements obtained within the first 24 hours after the onset of severe hyponatremia.

### Patient Course and Outcomes

**Figure 2:**
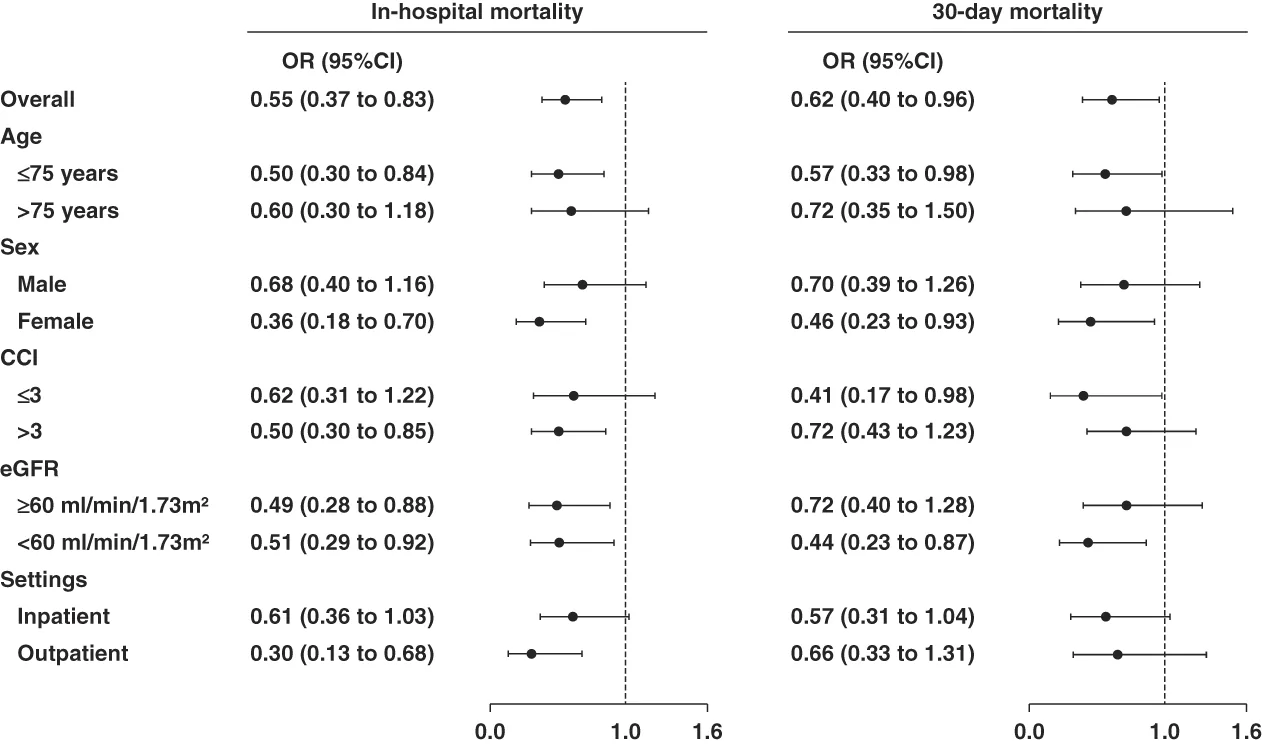
**Subgroup analyses of the effect of nephrology consultation on mortality.** Forest plot shows ORs and 95% CIs for in-hospital and 30-day mortality associated with nephrology consultation in various prespecified patient subgroups. Models were adjusted using IPTW. CI, confidence interval; CCI, Charlson Comorbidity Index; IPTW, inverse probability of treatment weighting; OR, odds ratio.

The clinical course and outcomes of patients with severe hyponatremia are presented in Table 3. With data on in-hospital and 30-day mortality available for all included patients, the nephrology consultation group showed lower in-hospital mortality (10% versus 23%, *P* < 0.001), lower 30-day mortality (8% versus 18%, *P* < 0.001), and shorter length of hospital stay (14 versus 19 days, *P* = 0.009). ODS occurred in only two patients in the nephrology consultation group and one in the non-nephrology consultation group, with no significant difference in incidence between the groups (*P* = 0.27). As shown in Table 4, unadjusted logistic regression (Model 1) demonstrated that nephrology consultation was associated with a reduced risk of in-hospital mortality (OR, 0.35; 95% CI, 0.23 to 0.53). In the fully adjusted multivariable model 3, nephrology consultation remained a significant predictor of favorable outcomes (OR, 0.46; 95% CI, 0.28 to 0.74). This relationship was consistent in the IPTW-adjusted model (OR, 0.55; 95% CI, 0.37 to 0.83). With regard to 30-day mortality, unadjusted analysis (model 1) showed a significant association between nephrology consultation and a reduced risk (OR, 0.40; 95% CI, 0.26 to 0.62), which persisted in both the fully adjusted Model 3 (OR, 0.53; 95% CI, 0.31 to 0.89) and the IPTW model (OR, 0.62; 95% CI, 0.40 to 0.96). Furthermore, the beneficial effect of nephrology consultation on mortality was generally consistent across subgroups by age, sex, CCI, eGFR, and clinical setting (Figure 2). The 24-hour change in serum sodium was 5.4 (2.5–8.9) mEq/L in the nephrology consultation group and 3.8 (1.0–7.2) mEq/L in the non-nephrology consultation group (*P* < 0.001). The proportions of patients experiencing slow, optimal, and fast correction were 55%, 27%, and 18% in the nephrology consultation group and 68%, 18%, and 14% in the non-nephrology consultation group, respectively (overall *P* < 0.001).

**Table 3:** Clinical outcomes in patients with severe hyponatremia stratified by nephrology consultation

Outcomes	Total (*N*=942)	Non-nephrology Consultation Group (*n*=600)	Nephrology Consultation Group (*n*=342)	*P* Value
**Changes in serum sodium level in 24 hours**				
Median (IQR), mEq/L	4.5 (1.5–7.7)	3.8 (1.0–7.2)	5.4 (2.5–8.9)	<0.001
Slow correction	597 (63%)	409 (68%)	188 (55%)	<0.001
Optimal correction	201 (21%)	108 (18%)	93 (27%)	
Fast correction	144 (15%)	83 (14%)	61 (18%)	
In-hospital mortality	173 (18%)	140 (23%)	33 (10%)	<0.001
30-d mortality	137 (15%)	109 (18%)	28 (8%)	<0.001
LOS, d	16 (9–31)	19 (10–34)	14 (9–27)	0.01
ODS	3 (0.3%)	1 (0.2%)	2 (0.6%)	0.27

Data are expressed as median (interquartile range) or number (percentage). Depending on the increase in serum sodium within 24 hours, correction was classified as slow (<6 mEq/L), optimal (6–10 mEq/L), or fast (>10 mEq/L).

IQR, interquartile range; LOS, length of hospital stay; ODS, osmotic demyelination syndrome.

**Table 4:** Logistic regression analysis of nephrology consultation on mortality outcomes

Model	In-Hospital Mortality		30-D Mortality	
	OR (95% CI)	*P* Value	OR (95% CI)	*P* Value
Model 1	0.35 (0.23 to 0.53)	<0.001	0.40 (0.26 to 0.62)	<0.001
Model 2	0.41 (0.27 to 0.63)	<0.001	0.46 (0.29 to 0.72)	<0.001
Model 3	0.46 (0.28 to 0.74)	0.001	0.53 (0.31 to 0.89)	0.02
IPTW-adjusted	0.55 (0.37 to 0.83)	0.004	0.62 (0.40 to 0.96)	0.03

Model 1: Unadjusted. Model 2: Adjusted for age, sex, and body mass index. Model 3: Further adjusted for setting in which hyponatremia occurs, Charlson Comorbidity Index, do-not-resuscitate order, eGFR, and sodium level at study inclusion.

CI, confidence interval; IPTW, inverse probability of treatment weighting; OR, odds ratio.

Six sensitivity analyses were conducted. First, in an analysis replacing CCI with individual comorbidities and laboratory parameters as covariates, nephrology consultation was associated with lower in-hospital mortality and showed a trend toward lower 30-day mortality (OR, 0.55; 95% CI, 0.33 to 0.93; and OR, 0.66; 95% CI, 0.38 to 1.16, respectively; Supplemental Table 1). Second, after excluding 120 patients with DNR order, nephrology consultation remained significantly associated with each mortality outcome (OR, 0.50; 95% CI, 0.29 to 0.86 and OR, 0.52; 95% CI, 0.28 to 0.97, respectively; Supplemental Table 2). Third, in the analysis restricted to the 822 patients managed in general wards, the main findings remained unchanged (Supplemental Table 3). Fourth, after excluding 93 patients with blood glucose >200 mg/dl, the association between nephrology consultation and lower mortality remained statistically significant (OR, 0.47; 95% CI, 0.29 to 0.78 and OR, 0.52; 95% CI, 0.30 to 0.90, respectively; Supplemental Table 4). Fifth, the sensitivity analysis using multiple imputation to replace missing values for body mass index and eGFR also yielded consistent results (Supplemental Table 5). Sixth, in a timing-stratified analysis using the non-nephrology consultation group as the reference, nephrology consultation within the first 24 hours was associated with lower mortality, whereas delayed nephrology consultation was not significantly associated with mortality (Supplemental Table 6).

## Discussion

This study investigated the association of early nephrology consultation with clinical outcomes in more than 900 patients with severe hyponatremia. We found that early nephrology consultation was significantly associated with lower in-hospital and 30-day mortality, as well as a greater 24-hour sodium correction. Our primary findings were consistent across subgroups and remained robust after sensitivity analyses.

The optimal treatment strategy for hyponatremia to improve prognosis remains unclear. Given that the risk of overly rapid correction of hyponatremia is well documented in clinical studies, both US and European guidelines advise limiting increases to no more than 10–12 mEq/L in 24 hours (no more than 8 mEq/L in patients at high risk for ODS) and 18 mEq/L in 48 hours to prevent ODS.^2,3^ On the other hand, the minimum correction rate has not been clearly described in current guidelines. Recent studies have demonstrated that optimal or rapid correction improved outcomes without elevating the risk of ODS, highlighting the potential significance of active management of hyponatremia.^12–15,23^ In this context, the utility of specific treatments, including hypertonic saline or vasopressin antagonists, has been examined; however, few studies have assessed the effect of specialist involvement.^10,12,18,24,25^ Nephrology consultation has been associated with improved outcomes in patients with AKI and CKD, presumably by enabling appropriate diagnosis, treatment, and monitoring.^16,17^ It remains an important question whether these favorable effects of nephrology consultation extend to the management of hyponatremia.

To our knowledge, this is the first study to indicate the association of nephrology consultation with lower in-hospital and 30-day mortality. In this study, the in-hospital and 30-day mortality rates were 18% and 15%, respectively, broadly consistent with previous reports.^13,19^ Patients who received nephrology consultation were more likely to receive hypertonic saline, undergo frequent laboratory monitoring, and consequently experience a higher rate of sodium correction—suggesting that nephrologists tend to use more active management strategies compared with non-nephrologists. Although the exact reasons for unfavorable outcomes in patients managed by non-nephrologists remain unclear, it is plausible that the rate of sodium correction played a role. Indeed, meta-analyses demonstrated that a low correction rate within 24 hours is associated with higher mortality and longer hospital stay.^13–15^ Several factors may account for the slower correction seen with non-nephrologist management. One is a limited awareness of the clinical relevance of prolonged hyponatremia because its often nonspecific symptoms might lead to underestimation of the associated risk. Another explanation is an undue fear of overcorrection of hyponatremia even in patients at low risk for ODS, which remains a serious iatrogenic complication. The complexity of individualized clinical assessment may incline nonspecialists toward a more conservative, risk-averse approach. In subgroup analyses, the association between nephrology consultation and lower mortality seemed more pronounced in female patients. In the context of potential sex-related differences in clinical susceptibility to hyponatremia, the underlying mechanisms of this observation remain an interesting topic for future research.^26^

This study has clinical implications. Hyponatremia is characterized by marked heterogeneity in patient background and treatment responsiveness but requires precise correction to avoid the risks of both overcorrection and insufficient correction—making its management particularly demanding. In this study, only 36% of patients received nephrology consultation, and this proportion seemed lower than that observed in AKI, another condition closely associated with patient prognosis.^17^ This might partly result from the absence of previous clinical studies or guidelines focusing on the specialty of the primary physician managing hyponatremia.^3,4^ We believe that our results could contribute to the broader implementation of nephrology consultation in the management of hyponatremia.

We acknowledge that this study has several limitations. Most importantly, this study was based on a retrospective design. Despite conducting IPTW-adjusted analyses, reverse causality and residual confounding due to unmeasured variables could not be fully excluded. Specifically, differences in patient background or illness severity, such as advanced cancer, heart failure, and cirrhosis, may have influenced the decision to involve nephrologists. In addition, in our health care setting, patients with severe hyponatremia are often initially managed by experienced subspecialists at tertiary hospitals, and nephrology consultation is readily available but not uniformly requested. Therefore, the decision to consult nephrologists may reflect perceived clinical complexity, uncertainty regarding management, or goals of care rather than serum sodium level alone.

To address this potential confounding, DNR order and CCI were included in the main adjusted analyses, and additional sensitivity analyses were performed in which individual comorbid conditions were included as covariates; these analyses yielded broadly consistent results. In addition, timing-stratified analyses showed that the association with lower mortality was evident mainly in patients receiving early consultation, while delayed consultation was not significantly associated with outcomes, supporting the notion that the timing of specialist involvement, rather than baseline patient characteristics alone, may be clinically meaningful, and suggesting that referral bias alone may not fully explain the observed associations. Confirmation of this causal relationship would require an interventional study, which may not be ethically feasible.

Furthermore, the longitudinal changes in kidney function and serum sodium levels prior to study inclusion could not be evaluated because nearly half of the patients were managed in the outpatient setting. Data extraction from electronic medical records was not blinded, which may have introduced information bias. In addition, we defined nephrology consultation as any involvement by nephrologists within 24 hours of the onset of severe hyponatremia. It is intriguing to consider time-dependent effects—whether even earlier intervention could further improve outcomes. This study was conducted at two tertiary care hospitals in Tokyo certified as educational facilities by the Japanese Society of Nephrology, and the potential for selection bias should be considered. Given that the role of nephrologists may vary across hospitals, regions, and countries, larger-scale studies are required to validate the generalizability of our main findings.

In conclusion, we demonstrated that early nephrology consultation was significantly associated with reduced in-hospital and 30-day mortality among patients with severe hyponatremia, highlighting the critical role of nephrologists in the management of this condition and the importance of interdisciplinary collaboration for optimizing patient outcomes.

## Supplementary Material

## Data Availability

Original data generated for the study will be made available upon reasonable request to the corresponding author. Data Type: Observational Data; Statistical Analysis Plan. Reason for Restricted Access: The data contain sensitive patient information and cannot be made publicly available due to ethical and legal restrictions. De-identified data may be shared upon reasonable request and subject to approval by the institutional review board and data use agreements.

## Declarative Statements

This study includes clinical experimentation and received Institutional Review Board or Ethics Committee approval. The need to obtain informed patient consent was waived. This study includes clinical experimentation and complies with the Declaration of Helsinki.

## Supplemental Material

Supplemental Table 1. Logistic regression analysis of nephrology consultation on mortality outcomes (replacing the CCI with individual comorbidities and incorporating additional disease severity indicators).

Supplemental Table 2. Logistic regression analysis of nephrology consultation on mortality outcomes (excluding patients with DNR order).

Supplemental Table 3. Logistic regression analysis of nephrology consultation on mortality outcomes (restricted to patients managed in general wards).

Supplemental Table 4. Logistic regression analysis of nephrology consultation on mortality outcomes (excluding patients with blood glucose >200 mg/dl).

Supplemental Table 5. Logistic regression analysis of nephrology consultation on mortality outcomes (using multiple imputation for missing values).

Supplemental Table 6. Logistic regression analysis of timing of nephrology consultation on mortality outcomes.
