# Supplementary material for: Association of Early Nephrology Consultation with Mortality in Patients with Severe Hyponatremia
Source: Kidney360. 2026 Apr 14;7(8):1763–71. doi: 10.34067/KID.0000001197 (PMC13567875; doi:10.34067/KID.0000001197)
Supplement: Supplementary file 1 [file kidney360-7-1763-s001.pdf]

# ASN Journal Disclosure Form

As per ASN journal policy, I have disclosed any financial relationships or commitments I have held in the past 36 months as included below. I have listed my Current Employer below to indicate there is a relationship requiring disclosure. If no relationship exists, my Current Employer is not listed.

T. Azegami reports the following:
Employer: Keio University School of Medicine; Research Funding: Mochida Pharmaceutical Co., Ltd.; Speakers Bureau: Received honoraria for speaking at company-sponsored meetings or seminars for the following companies: Otsuka Pharmaceutical Co., Ltd.; Mochida Pharmaceutical Co., Ltd.; Boehringer Ingelheim Japan, Ltd.; AstraZeneca K.K.; Mitsubishi Tanabe Pharma Corporation; Bayer Yakuhin, Ltd.; Novartis Pharma K.K.; Novo Nordisk Pharma Ltd.; Kowa Company, Ltd.; Taisho Pharmaceutical Co., Ltd.; Torii Pharmaceutical Co., Ltd.; Zeria Pharmaceutical Co., Ltd.; and Other Interests or Relationships: Member, Japanese Society of Nephrology; Japanese Society of Hypertension.

I understand that the information above will be published within the journal article, if accepted, and that failure to comply and/or to accurately and completely report the potential financial conflicts of interest could lead to the following: 1) Prior to publication, article rejection, or 2) Post-publication, sanctions ranging from, but not limited to, issuing a correction, reporting the inaccurate information to the authors' institution, banning authors from submitting work to ASN journals for varying lengths of time, and/or retraction of the published work.

Name: Tatsuhiko Azegami
Manuscript ID: K360-2025-001442R1
Manuscript Title: Association of Early Nephrology Consultation with Mortality in Patients with Severe Hyponatremia
Date of Completion: February 9, 2026
Disclosure Updated Date: February 9, 2026

ASN American Society of Nephrology | JASN Journal of the American Society of Nephrology | KIDNEY360 Accessing Our World From Every Angle | CJASN Clinical Journal of the American Society of Nephrology

# ASN Journal Disclosure Form

As per ASN journal policy, I have disclosed any financial relationships or commitments I have held in the past 36 months as included below. I have listed my Current Employer below to indicate there is a relationship requiring disclosure. If no relationship exists, my Current Employer is not listed.

K. Hayashi reports the following:
Employer: Keio University School of Medicine; Research Funding: Boehringer Ingelheim; Kyowa Hakko Kirin; Nisshin Shokuhin; Speakers Bureau: Boehringer Ingelheim, Kyowa Hakko Kirin, Novartis, Kowa Company, Ltd., Mitsubishi Tanabe Pharma Corporation, Astellas Pharma Inc. Ono Pharmaceutical Co., Ltd. Novo Nordisk Pharma Ltd.;; and Other Interests or Relationships: A member of Japan Kidney Disease Association and Japanese Association of Hypertension.

I understand that the information above will be published within the journal article, if accepted, and that failure to comply and/or to accurately and completely report the potential financial conflicts of interest could lead to the following: 1) Prior to publication, article rejection, or 2) Post-publication, sanctions ranging from, but not limited to, issuing a correction, reporting the inaccurate information to the authors' institution, banning authors from submitting work to ASN journals for varying lengths of time, and/or retraction of the published work.

Name: Kaori Hayashi
Manuscript ID: K360-2025-001442R2
Manuscript Title: Association of Early Nephrology Consultation with Mortality in Patients with Severe Hyponatremia
Date of Completion: March 7, 2026
Disclosure Updated Date: February 9, 2026

ASN American Society of Nephrology
JASN Journal of the American Society of Nephrology
KIDNEY360 Accessing Our World From Every Angle
CJASN Clinical Journal of the American Society of Nephrology

# ASN Journal Disclosure Form

As per ASN journal policy, I have disclosed any financial relationships or commitments I have held in the past 36 months as included below. I have listed my Current Employer below to indicate there is a relationship requiring disclosure. If no relationship exists, my Current Employer is not listed.

A. Hagiwara reports the following:
Employer: Keio university

I understand that the information above will be published within the journal article, if accepted, and that failure to comply and/or to accurately and completely report the potential financial conflicts of interest could lead to the following: 1) Prior to publication, article rejection, or 2) Post-publication, sanctions ranging from, but not limited to, issuing a correction, reporting the inaccurate information to the authors' institution, banning authors from submitting work to ASN journals for varying lengths of time, and/or retraction of the published work.

Name: Aika Hagiwara
Manuscript ID: K360-2025-001442R4
Manuscript Title: Association of Early Nephrology Consultation with Mortality in Patients with Severe Hyponatremia
Date of Completion: April 8, 2026
Disclosure Updated Date: March 11, 2026

ASN JASN Journal of the American Society of Nephrology KIDNEY360 Accessing Our World From Every Angle CJASN Clinical Journal of the American Society of Nephrology

# ASN Journal Disclosure Form

As per ASN journal policy, I have disclosed any financial relationships or commitments I have held in the past 36 months as included below. I have listed my Current Employer below to indicate there is a relationship requiring disclosure. If no relationship exists, my Current Employer is not listed.

A. Hishikawa has nothing to disclose.

I understand that the information above will be published within the journal article, if accepted, and that failure to comply and/or to accurately and completely report the potential financial conflicts of interest could lead to the following: 1) Prior to publication, article rejection, or 2) Post-publication, sanctions ranging from, but not limited to, issuing a correction, reporting the inaccurate information to the authors' institution, banning authors from submitting work to ASN journals for varying lengths of time, and/or retraction of the published work.

Name: Akihito Hishikawa
Manuscript ID: K360-2025-001442R3
Manuscript Title: Association of Early Nephrology Consultation with Mortality in Patients with Severe Hyponatremia
Date of Completion: March 11, 2026
Disclosure Updated Date: March 11, 2026

ASN American Society of Nephrology | JASN Journal of the American Society of Nephrology | KIDNEY360 Accessing Our World From Every Angle | CJASN Clinical Journal of the American Society of Nephrology

# ASN Journal Disclosure Form

As per ASN journal policy, I have disclosed any financial relationships or commitments I have held in the past 36 months as included below. I have listed my Current Employer below to indicate there is a relationship requiring disclosure. If no relationship exists, my Current Employer is not listed.

M. Komatsu has nothing to disclose.

I understand that the information above will be published within the journal article, if accepted, and that failure to comply and/or to accurately and completely report the potential financial conflicts of interest could lead to the following: 1) Prior to publication, article rejection, or 2) Post-publication, sanctions ranging from, but not limited to, issuing a correction, reporting the inaccurate information to the authors' institution, banning authors from submitting work to ASN journals for varying lengths of time, and/or retraction of the published work.

Name: Motoaki Komatsu
Manuscript ID: K360-2025-001442R1
Manuscript Title: Association of Early Nephrology Consultation with Mortality in Patients with Severe Hyponatremia
Date of Completion: February 10, 2026
Disclosure Updated Date: September 4, 2025

ASN American Society of Nephrology
JASN Journal of the American Society of Nephrology
KIDNEY360 Accessing Our World From Every Angle
CJASN Clinical Journal of the American Society of Nephrology

# ASN Journal Disclosure Form

As per ASN journal policy, I have disclosed any financial relationships or commitments I have held in the past 36 months as included below. I have listed my Current Employer below to indicate there is a relationship requiring disclosure. If no relationship exists, my Current Employer is not listed.

R. Mitsuno reports the following:
Employer: Keio University

I understand that the information above will be published within the journal article, if accepted, and that failure to comply and/or to accurately and completely report the potential financial conflicts of interest could lead to the following: 1) Prior to publication, article rejection, or 2) Post-publication, sanctions ranging from, but not limited to, issuing a correction, reporting the inaccurate information to the authors' institution, banning authors from submitting work to ASN journals for varying lengths of time, and/or retraction of the published work.

Name: Ryunosuke Mitsuno
Manuscript ID: K360-2025-001442R1
Manuscript Title: Association of Early Nephrology Consultation with Mortality in Patients with Severe Hyponatremia.
Date of Completion: February 7, 2026
Disclosure Updated Date: February 7, 2026

# ASN Journal Disclosure Form

As per ASN journal policy, I have disclosed any financial relationships or commitments I have held in the past 36 months as included below. I have listed my Current Employer below to indicate there is a relationship requiring disclosure. If no relationship exists, my Current Employer is not listed.

H. Miyauchi has nothing to disclose.

I understand that the information above will be published within the journal article, if accepted, and that failure to comply and/or to accurately and completely report the potential financial conflicts of interest could lead to the following: 1) Prior to publication, article rejection, or 2) Post-publication, sanctions ranging from, but not limited to, issuing a correction, reporting the inaccurate information to the authors' institution, banning authors from submitting work to ASN journals for varying lengths of time, and/or retraction of the published work.

Name: Hiroaki Miyauchi
Manuscript ID: K360-2025-001442R1
Manuscript Title: Association of Early Nephrology Consultation with Mortality in Patients with Severe Hyponatremia
Date of Completion: February 12, 2026
Disclosure Updated Date: February 12, 2026

ASN
American Society of Nephrology
JASN
Journal of the American Society of Nephrology
KIDNEY360
Accessing Our World From Every Angle
CJASN
Clinical Journal of the American Society of Nephrology

# ASN Journal Disclosure Form

As per ASN journal policy, I have disclosed any financial relationships or commitments I have held in the past 36 months as included below. I have listed my Current Employer below to indicate there is a relationship requiring disclosure. If no relationship exists, my Current Employer is not listed.

A. Nakamura reports the following:
Employer: Keio University School of Medicine

I understand that the information above will be published within the journal article, if accepted, and that failure to comply and/or to accurately and completely report the potential financial conflicts of interest could lead to the following: 1) Prior to publication, article rejection, or 2) Post-publication, sanctions ranging from, but not limited to, issuing a correction, reporting the inaccurate information to the authors' institution, banning authors from submitting work to ASN journals for varying lengths of time, and/or retraction of the published work.

Name: Akira Nakamura
Manuscript ID: K360-2025-001442R3
Manuscript Title: Association of Early Nephrology Consultation with Mortality in Patients with Severe Hyponatremia
Date of Completion: March 15, 2026
Disclosure Updated Date: March 15, 2026

ASN American Society of Nephrology | JASN Journal of the American Society of Nephrology | KIDNEY360 Accessing Our World From Every Angle | CJASN Clinical Journal of the American Society of Nephrology

# ASN Journal Disclosure Form

As per ASN journal policy, I have disclosed any financial relationships or commitments I have held in the past 36 months as included below. I have listed my Current Employer below to indicate there is a relationship requiring disclosure. If no relationship exists, my Current Employer is not listed.

K. Nakamura reports the following:
Employer: Keio University Hospital

I understand that the information above will be published within the journal article, if accepted, and that failure to comply and/or to accurately and completely report the potential financial conflicts of interest could lead to the following: 1) Prior to publication, article rejection, or 2) Post-publication, sanctions ranging from, but not limited to, issuing a correction, reporting the inaccurate information to the authors' institution, banning authors from submitting work to ASN journals for varying lengths of time, and/or retraction of the published work.

Name: Kyosei Nakamura
Manuscript ID: K360-2025-001442R3
Manuscript Title: Association of Early Nephrology Consultation with Mortality in Patients with Severe Hyponatremia
Date of Completion: March 13, 2026
Disclosure Updated Date: March 13, 2026

ASN
American Society of Nephrology
JASN
Journal of the American Society of Nephrology
KIDNEY360
Accessing Our World From Every Angle
CJASN
Clinical Journal of the American Society of Nephrology

# ASN Journal Disclosure Form

As per ASN journal policy, I have disclosed any financial relationships or commitments I have held in the past 36 months as included below. I have listed my Current Employer below to indicate there is a relationship requiring disclosure. If no relationship exists, my Current Employer is not listed.

T. Nakayama has nothing to disclose.

I understand that the information above will be published within the journal article, if accepted, and that failure to comply and/or to accurately and completely report the potential financial conflicts of interest could lead to the following: 1) Prior to publication, article rejection, or 2) Post-publication, sanctions ranging from, but not limited to, issuing a correction, reporting the inaccurate information to the authors' institution, banning authors from submitting work to ASN journals for varying lengths of time, and/or retraction of the published work.

Name: Takashin Nakayama
Manuscript ID: K360-2025-001442R3
Manuscript Title: Association of Early Nephrology Consultation with Mortality in Patients with Severe Hyponatremia
Date of Completion: March 11, 2026
Disclosure Updated Date: March 11, 2026

ASN American Society of Nephrology
JASN Journal of the American Society of Nephrology
KIDNEY360 Accessing Our World From Every Angle
CJASN Clinical Journal of the American Society of Nephrology

# ASN Journal Disclosure Form

As per ASN journal policy, I have disclosed any financial relationships or commitments I have held in the past 36 months as included below. I have listed my Current Employer below to indicate there is a relationship requiring disclosure. If no relationship exists, my Current Employer is not listed.

S. Sugihara has nothing to disclose.

I understand that the information above will be published within the journal article, if accepted, and that failure to comply and/or to accurately and completely report the potential financial conflicts of interest could lead to the following: 1) Prior to publication, article rejection, or 2) Post-publication, sanctions ranging from, but not limited to, issuing a correction, reporting the inaccurate information to the authors' institution, banning authors from submitting work to ASN journals for varying lengths of time, and/or retraction of the published work.

Name: Shinnosuke Sugihara
Manuscript ID: K360-2025-001442R2
Manuscript Title: Association of Early Nephrology Consultation with Mortality in Patients with Severe Hyponatremia
Date of Completion: March 13, 2026
Disclosure Updated Date: March 13, 2026

ASN American Society of Nephrology
JASN Journal of the American Society of Nephrology
KIDNEY360 Accessing Our World From Every Angle
CJASN Clinical Journal of the American Society of Nephrology

# ASN Journal Disclosure Form

As per ASN journal policy, I have disclosed any financial relationships or commitments I have held in the past 36 months as included below. I have listed my Current Employer below to indicate there is a relationship requiring disclosure. If no relationship exists, my Current Employer is not listed.

N. Yoshimoto reports the following:
Employer: Keio University School of Medicine

I understand that the information above will be published within the journal article, if accepted, and that failure to comply and/or to accurately and completely report the potential financial conflicts of interest could lead to the following: 1) Prior to publication, article rejection, or 2) Post-publication, sanctions ranging from, but not limited to, issuing a correction, reporting the inaccurate information to the authors' institution, banning authors from submitting work to ASN journals for varying lengths of time, and/or retraction of the published work.

Name: Norifumi Yoshimoto
Manuscript ID: K360-2025-001442R1
Manuscript Title: Association of Early Nephrology Consultation with Mortality in Patients with Severe Hyponatremia
Date of Completion: February 9, 2026
Disclosure Updated Date: February 9, 2026
