# Supplementary material for: Association of Early Nephrology Consultation with Mortality in Patients with Severe Hyponatremia
Source: Kidney360. 2026 Apr 14;7(8):1763–71. doi: 10.34067/KID.0000001197 (PMC13567875; doi:10.34067/KID.0000001197)
Supplement: Supplementary file 2 [file kidney360-7-1763-s002.pdf]

**Supplemental Material**

# Association of Early Nephrology Consultation with Mortality in Patients with Severe Hyponatremia

Akira Nakamura[1*], Takashin Nakayama[1*], Tatsuhiko Azegami[1], Kyosei Nakamura[1], Hiroaki Miyauchi[1], Shinnosuke Sugihara[1], Ryunosuke Mitsuno[1], Norifumi Yoshimoto[1], Akihito Hishikawa[1], Aika Hagiwara[1], Motoaki Komatsu[2], Kaori Hayashi[1]

1 Division of Nephrology, Endocrinology, and Metabolism, Department of Internal Medicine, Keio University School of Medicine, Tokyo, Japan.
2 Department of Nephrology, Tokyo Saiseikai Central Hospital, Tokyo, Japan.
*These authors contributed equally to this work.

## Table of Contents

**Supplemental Table 1.** Logistic regression analysis of nephrology consultation on mortality outcomes (replacing the Charlson comorbidity index with individual comorbidities and incorporating additional disease severity indicators)

**Supplemental Table 2.** Logistic regression analysis of nephrology consultation on mortality outcomes (excluding patients with do-not-resuscitate order)

**Supplemental Table 3.** Logistic regression analysis of nephrology consultation on mortality outcomes (restricted to patients managed in general wards)

**Supplemental Table 4.** Logistic regression analysis of nephrology consultation on mortality outcomes (excluding patients with blood glucose >200 mg/dL)

**Supplemental Table 5.** Logistic regression analysis of nephrology consultation on mortality outcomes (using multiple imputation for missing values)

**Supplemental Table 6.** Logistic regression analysis of timing of nephrology consultation on mortality outcomes

**Supplemental Table 1. Logistic regression analysis of nephrology consultation on mortality outcomes (replacing the Charlson comorbidity index with individual comorbidities and incorporating additional disease severity indicators)**

| | In-hospital mortality | | 30-day mortality | |
|---|---|---|---|---|
| | OR (95%CI) | *P* value | OR (95%CI) | *P* value |
| Model 1 | 0.35 (0.23–0.53) | < 0.001 | 0.40 (0.26–0.62) | < 0.001 |
| Model 2 | 0.41 (0.27–0.63) | < 0.001 | 0.46 (0.29–0.72) | < 0.001 |
| Model 3 | 0.55 (0.33–0.93) | 0.03 | 0.66 (0.38–1.16) | 0.15 |

Model 1: Unadjusted. Model 2: Adjusted for age, sex and body mass index. Model 3: Further adjusted for setting in which hyponatremia occurs, do-not-resuscitate order, heart failure, cirrhosis, metastatic tumor, sepsis, estimated glomerular filtration rate, sodium level, albumin, hemoglobin at study inclusion. Abbreviations: OR, odds ratio; CI, confidence interval.

**Supplemental Table 2. Logistic regression analysis of nephrology consultation on mortality outcomes (excluding patients with do-not-resuscitate order)**

| | In-hospital mortality | | 30-day mortality | |
|---|---|---|---|---|
| | OR (95%CI) | *P* value | OR (95%CI) | *P* value |
| Model 1 | 0.42 (0.26–0.68) | < 0.001 | 0.46 (0.27–0.79) | 0.005 |
| Model 2 | 0.50 (0.31–0.83) | 0.008 | 0.53 (0.30–0.94) | 0.03 |
| Model 3 | 0.50 (0.29–0.86) | 0.01 | 0.52 (0.28–0.97) | 0.04 |

Model 1: Unadjusted. Model 2: Adjusted for age, sex and body mass index. Model 3: Further adjusted for setting in which hyponatremia occurs, Charlson comorbidity index, estimated glomerular filtration rate, and sodium level at study inclusion.
Abbreviations: OR, odds ratio; CI, confidence interval.

**Supplemental Table 3. Logistic regression analysis of nephrology consultation on mortality outcomes (restricted to patients managed in general wards)**

| | In-hospital mortality | | 30-day mortality | |
|---|---|---|---|---|
| | OR (95%CI) | *P* value | OR (95%CI) | *P* value |
| Model 1 | 0.38 (0.25–0.58) | < 0.001 | 0.45 (0.28–0.71) | < 0.001 |
| Model 2 | 0.43 (0.28–0.68) | < 0.001 | 0.50 (0.31–0.81) | 0.005 |
| Model 3 | 0.47 (0.28–0.78) | 0.003 | 0.57 (0.33–0.99) | 0.048 |

Model 1: Unadjusted. Model 2: Adjusted for age, sex and body mass index. Model 3: Further adjusted for setting in which hyponatremia occurs, Charlson comorbidity index, do-not-resuscitate order, estimated glomerular filtration rate, and sodium level at study inclusion.
Abbreviations: OR, odds ratio; CI, confidence interval.

**Supplemental Table 4. Logistic regression analysis of nephrology consultation on mortality outcomes (excluding patients with blood glucose >200 mg/dL)**

| | In-hospital mortality | | 30-day mortality | |
|---|---|---|---|---|
| | OR (95%CI) | *P* value | OR (95%CI) | *P* value |
| Model 1 | 0.35 (0.23–0.53) | < 0.001 | 0.39 (0.24–0.62) | < 0.001 |
| Model 2 | 0.41 (0.26–0.64) | < 0.001 | 0.44 (0.27–0.71) | < 0.001 |
| Model 3 | 0.47 (0.29–0.78) | 0.003 | 0.52 (0.30–0.90) | 0.02 |

Model 1: Unadjusted. Model 2: Adjusted for age, sex and body mass index. Model 3: Further adjusted for setting in which hyponatremia occurs, Charlson comorbidity index, do-not-resuscitate order, estimated glomerular filtration rate, and sodium level at study inclusion.
Abbreviations: OR, odds ratio; CI, confidence interval.

**Supplemental Table 5. Logistic regression analysis of nephrology consultation on mortality outcomes (using multiple imputation for missing values)**

| | In-hospital mortality | | 30-day mortality | |
|---|---|---|---|---|
| | OR (95%CI) | *P* value | OR (95%CI) | *P* value |
| Model 1 | 0.35 (0.23–0.53) | < 0.001 | 0.40 (0.26–0.62) | < 0.001 |
| Model 2 | 0.34 (0.22–0.51) | < 0.001 | 0.39 (0.25–0.61) | < 0.001 |
| Model 3 | 0.38 (0.24–0.60) | < 0.001 | 0.46 (0.28–0.74) | 0.002 |

Model 1: Unadjusted. Model 2: Adjusted for age, sex and body mass index. Model 3: Further adjusted for setting in which hyponatremia occurs, Charlson comorbidity index, do-not-resuscitate order, estimated glomerular filtration rate, and sodium level at study inclusion.
Abbreviations: OR, odds ratio; CI, confidence interval.

**Supplemental Table 6. Logistic regression analysis of timing of nephrology consultation on mortality outcomes**

| | | In-hospital mortality | | 30-day mortality | |
|---|---|---|---|---|---|
| | | OR (95%CI) | *P* value | OR (95%CI) | *P* value |
| Model 1 | Nephrology consultation | 0.34 (0.22–0.51) | < 0.001 | 0.38 (0.25–0.59) | < 0.001 |
| | Delayed nephrology consultation | 0.59 (0.27–1.28) | 0.18 | 0.47 (0.18–1.20) | 0.11 |
| Model 2 | Nephrology consultation | 0.40 (0.26–0.62) | < 0.001 | 0.44 (0.28–0.70) | < 0.001 |
| | Delayed nephrology consultation | 0.75 (0.34–1.66) | 0.47 | 0.56 (0.21–1.46) | 0.24 |
| Model 3 | Nephrology consultation | 0.45 (0.28–0.74) | 0.001 | 0.51 (0.30–0.86) | 0.01 |
| | Delayed nephrology consultation | 0.93 (0.38–2.30) | 0.88 | 0.67 (0.23–2.00) | 0.47 |

Reference category: no nephrology consultation throughout hospitalization. Nephrology consultation indicates consultation occurring within 24 hours from the onset of severe hyponatremia; delayed nephrology consultation indicates consultation occurring thereafter. Model 1: Unadjusted. Model 2: Adjusted for age, sex and body mass index. Model 3: Further adjusted for setting in which hyponatremia occurs, Charlson comorbidity index, do-not-resuscitate order, estimated glomerular filtration rate, and sodium level at study inclusion. Abbreviations: OR, odds ratio; CI, confidence interval.
